# Primary Enterolithiasis Associated With Severe Hypothyroidism and Ileal Stricture Presenting As Small Bowel Obstruction: A Case Report

**DOI:** 10.7759/cureus.107001

**Published:** 2026-04-13

**Authors:** Shantanu Tyagi, Ajay Kumar Dhiman, Mohim Thakur, Manish Kumar, Chongtham Anil Kumar Singh

**Affiliations:** 1 General Surgery, All India Institute of Medical Sciences, Bilaspur, Bilaspur, IND

**Keywords:** early surgical exploration, hypothyroidism, ileal stricture, primary enterolith, subacute intestinal obstruction

## Abstract

Enterolithiasis is an uncommon condition characterized by the formation of stones within the gastrointestinal tract and may rarely present as small bowel obstruction, typically developing in areas of intestinal stasis such as strictures, diverticula, or blind loops. Severe hypothyroidism can contribute to intestinal dysmotility and predispose to such stasis. We report a case of a 76-year-old woman who presented with abdominal pain, constipation, vomiting, and inability to pass stool or flatus for four days. Imaging demonstrated dilated small bowel loops with an intraluminal radio-opaque lesion consistent with an enterolith, and laboratory evaluation revealed severe primary hypothyroidism. An exploratory laparotomy identified an enterolith of size 2 x 3 cm in the proximal ileum just proximal to an ileal stricture. Enterotomy with stone extraction along with stricturoplasty was performed. The postoperative course was uneventful, and the patient was initiated on thyroid hormone replacement therapy. Severe hypothyroidism should be considered a potential contributory factor in enterolithiasis, and surgical intervention should address both the enterolith and the underlying stricture.

## Introduction

Subacute intestinal obstruction is a frequently encountered surgical emergency. Its primary causes include strictures, polyps, and tumors, with gallstones or foreign bodies being less common culprits [1]. Enteroliths, although rare, can also cause intestinal obstruction.

Enterolithiasis, i.e., the presence of stones within the gastrointestinal (GI) tract, is a rare condition with an incidence of 0.3%-10%, reflecting variations across different study populations, including autopsy series and clinical reports. Enteroliths are formed within areas of stasis due to various conditions. They can be primary or secondary and true or false, can cause obstruction and perforation, or may be asymptomatic. Enteroliths typically form near strictures, within diverticula, or in blind loops, often due to stasis [2].

Severe hypothyroidism may also present with small bowel obstruction due to intestinal dysmotility, adynamic ileus, and pseudo-obstruction. The literature describes very few case reports where patients with hypothyroidism present with small bowel obstruction without any mechanical obstruction and symptoms improved after thyroid replacement therapy [3,4]. However, the combination of hypothyroidism-induced intestinal dysmotility and a mechanical stricture may synergistically promote enterolith formation.

## Case presentation

A 76-year-old woman, a resident of Mandi district of Himachal Pradesh, visited our emergency department with complaints of inability to pass stool or flatus for four days, along with nausea and vomiting episodes for the past one day. These symptoms were associated with dull aching diffuse abdominal pain, which was continuous, not relieved by medications. The patient had long-standing constipation (15 years), generally relieved with laxatives every two to three days, but the current episode was refractory to medical management.

She was moderately built with coarse facies and dehydrated, and had dry skin with reduced skin turgor. Abdominal examination revealed a distended abdomen with no gross organomegaly, and bowel sounds were heard in all quadrants, but more in the right lower quadrant. Rectal examination showed fecal staining without gross anomalies. Basic blood tests were performed, and plain radiographs were requested to determine any obstruction. The thyroid profile was also evaluated as the patient had a history of chronic constipation.

**Figure 1:**
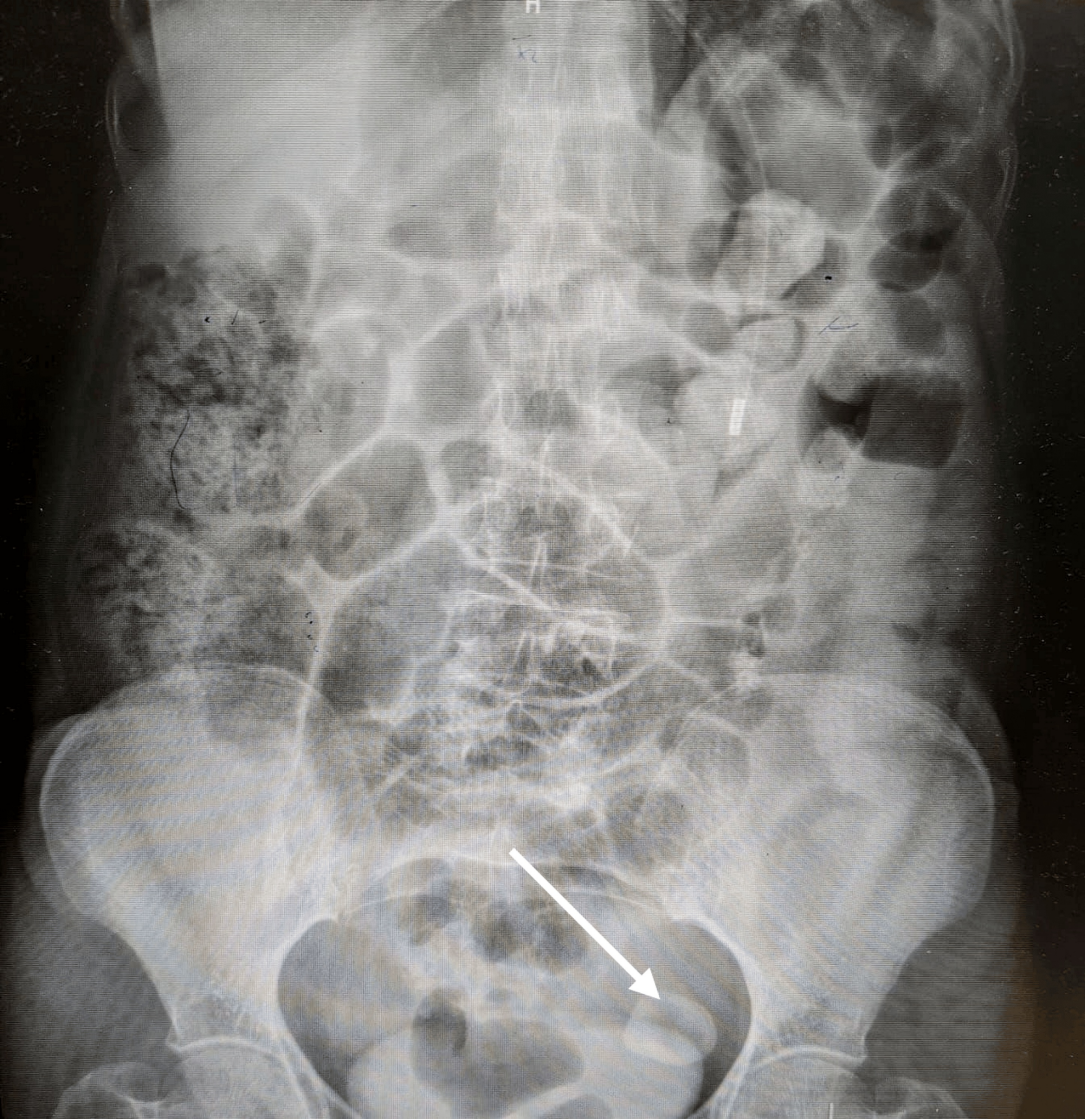
Plain abdominal radiograph demonstrating dilated small bowel loops with a radio-opaque intraluminal calculus (arrow), suggestive of an enterolith.

Hemogram revealed mild anemia with a normal leucocyte count. Liver and kidney function tests also showed normal findings. Thyroid profile revealed severe primary hypothyroidism (triiodothyronine (T3): 0.2 nmol/L (1.2-3.1 nmol/L), thyroxine (T4): 0.48 nmol/L (60-150 nmol/L), and thyroid-stimulating hormone (TSH): 67.08 mIU/L (0.4-4.0 mIU/L)), and plain radiographs demonstrated dilated small bowel loops with stone-like structures in the pelvis (Figure 1).

**Figure 2:**
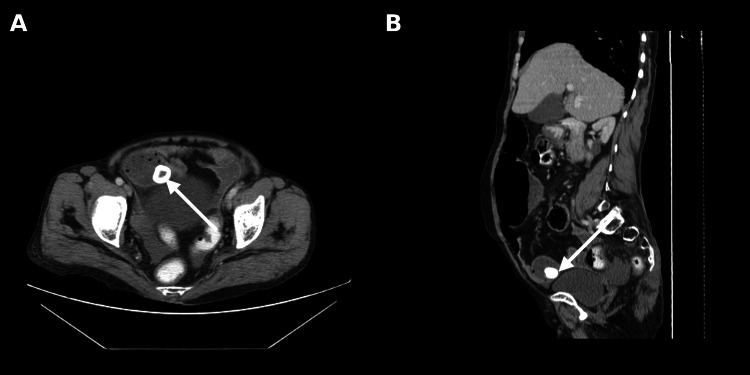
(A) Axial section showing a well-defined hyperdense calculus (arrow) located within a loop of the distal ileum. (B) Sagittal reconstruction confirming the intraluminal location of the calculus (arrow) in the distal ileum.

Immediately, whole-abdomen contrast-enhanced computed tomography (CECT) was performed, which confirmed dilated small bowel loops (distal jejunum and proximal ileum) with enteroliths in the distal portion of the dilated small bowel loops, along with normal loops in the distal ileal region (Figure 2). Abdominal ultrasound revealed antral gastritis with minimal free fluid in the pelvis and no evidence of gallstones.

**Figure 3:**
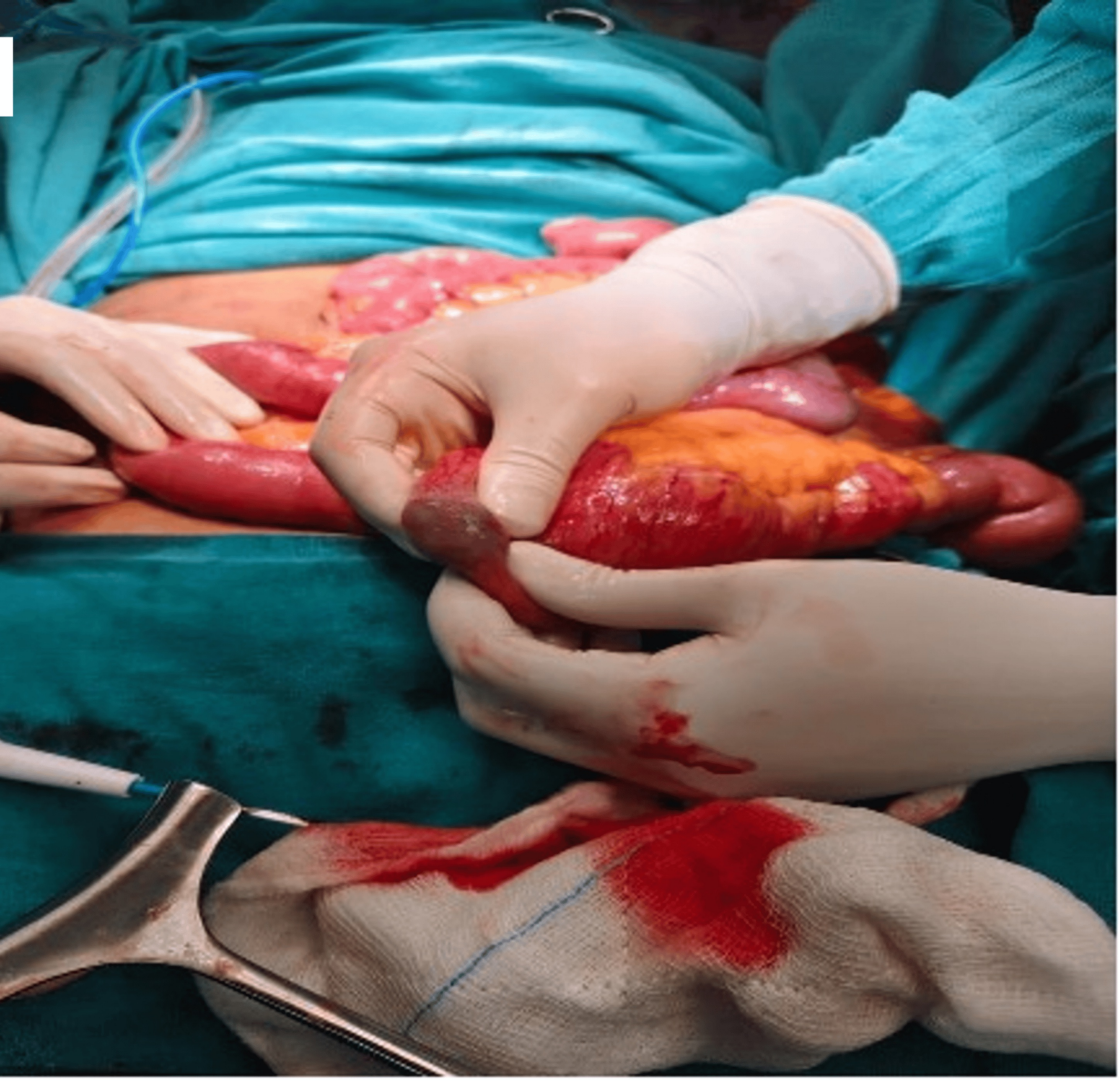
Intraoperative image demonstrating an intraluminal calculus within a distal ileal loop.

Next, an emergency exploratory laparotomy was planned. Endocrine opinion was consulted for severe hypothyroidism, and surgery was planned under steroid treatment along with T3 supplementation and consideration of the possible risk of myxedema coma. Intra-operatively, dilated ileal loops and an enterolith measuring 2 × 3 cm in the proximal ileal loops just proximal to a stricture around 130 cm proximal to the ileocecal junction were observed (Figure 3).

**Figure 4:**
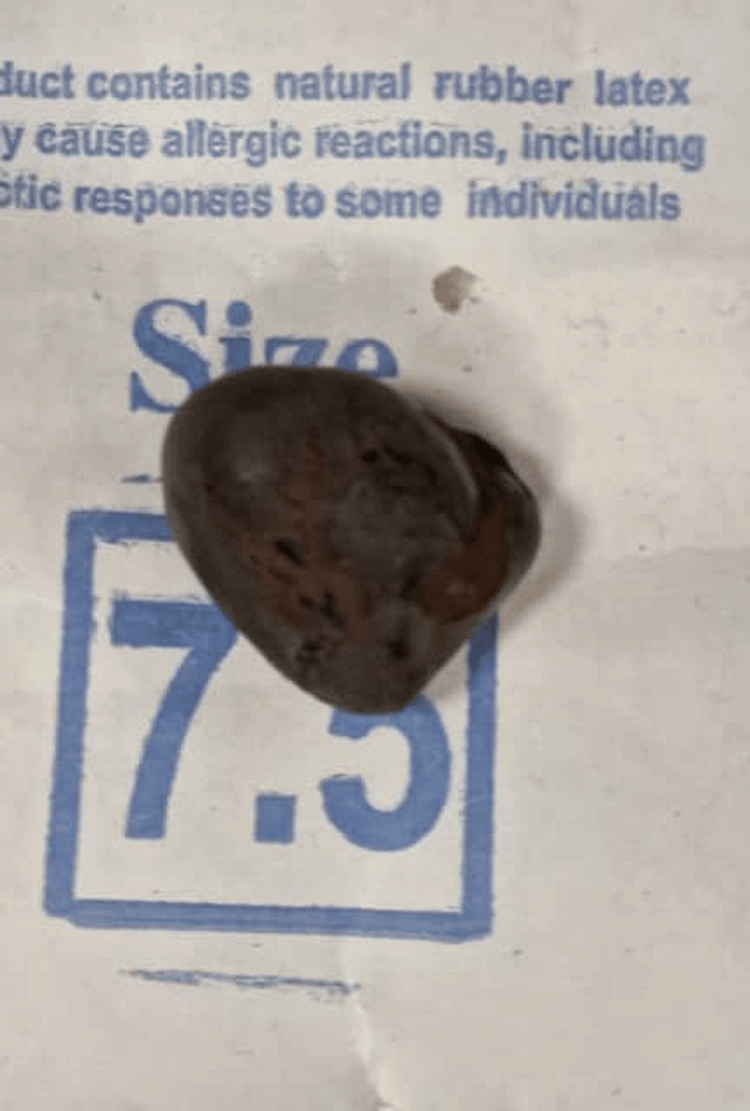
Gross specimen of the extracted intraluminal calculus from the distal ileum, appearing as a dark brown, smooth-surfaced stone consistent with an enterolith.

Enterotomy with enterolith extraction along with stricturoplasty was performed. A single stone measuring approximately 2 × 3 cm was extracted (Figure 4). Fecaliths were also detected in the descending colon.

Postoperatively, the patient was placed under close monitoring by the endocrinology team. Tests for anti-thyroid peroxidase (anti-TPO) antibodies revealed negative results, the steroid therapy was gradually tapered off, and the patient was started on levothyroxine supplementation. The patient started passing stool and flatus from postoperative day 4 onward and resumed the oral diet. She was discharged on postoperative day 6 with stable vitals, oral feed tolerance, passing flatus and feces, a healthy midline wound, and on levothyroxine supplementation with advice to undergo Endocrinology OPD and General Surgery follow-up.

## Discussion

Enterolithiasis, defined as the presence of GI concretions, is a rare condition that predominantly affects adults. Enteroliths vary in shape and size, ranging from a few millimeters to several centimeters, and can present as a solitary stone or a group of concretions. Enteroliths can be primary or secondary depending on the site of origin. Primary enteroliths originate within the GI tract, generally in anatomically abnormal regions such as diverticula, stricture, and blind loops, and are further subdivided into true and false enteroliths [5]. True enteroliths develop from the substance already present in the intestine, whereas false enteroliths develop around a foreign nidus [6]. Local factors such as intestinal dysmotility, luminal stasis, segment-specific pH, and the intestinal microenvironment play a pivotal role in the formation of enteroliths [7].

Secondary enteroliths originate outside the intestine and migrate intraluminally, most commonly as gallbladder stones through a cholecystoenteric fistula, presenting as a gallstone ileus [8]. Stones >20 mm typically cause obstruction, most often in the distal ileum [9].

This case emphasizes the diagnostic dilemma posed by enterolithiasis in a severely hypothyroid patient with an associated stricture resulting in obstruction. Studies suggest hypothyroidism as a risk factor for enterolith formation, probably due to slowed GI motility; however, there is no specific, quantifiable incidence rate linking the two conditions directly in the general population. Intestinal stricture and obstruction are rare but serious complications of severe, untreated, or poorly managed hypothyroidism; they are generally described in isolated cases. Severe hypothyroidism is known to reduce GI motility, leading to prolonged intestinal transit and stasis. This environment favors precipitation and aggregation of luminal contents, facilitating enterolith formation. When combined with a structural abnormality such as an ileal stricture, the effect is amplified, creating a localized environment highly conducive to stone formation [3,4].

Intermittent or "tumbling" symptoms occur as the enterolith migrates through the bowel lumen, producing intermittent and partial obstruction [1]. Detailed history-taking and physical examination with radiological investigation are required to establish the diagnosis in at-risk cases [10]. Reported complications include bowel obstruction, pressure necrosis, and GI hemorrhage and perforation [1]. Mortality rates have been reported at up to 3% in primary enterolithiasis and up to 8% in secondary enterolithiasis, particularly in a patient with significant obstruction and delayed diagnosis [2]. Differential diagnoses in such cases include gallstone ileus, bezoar, foreign body, and neoplastic obstruction, all of which must be considered and excluded through appropriate imaging and intraoperative assessment.

Management generally involves only a surgical procedure [9]. Initial intraoperative measures may include gentle crushing and milking of stones in the colon; however, enterotomy or segmental resection is necessary if this approach fails or some underlying pathology is present [10]. In our case, surgical intervention was warranted due to complete obstruction and the presence of a fixed mechanical cause. Enterotomy with stone extraction and stricturoplasty addressed both the obstructing enterolith and the underlying pathology, thereby reducing the risk of recurrence.

Our case has some limitations that we should acknowledge. Stone composition analysis was not performed, which limits the precise classification of the enterolith. Long-term follow-up data regarding thyroid function normalization could not be fully evaluated.

## Conclusions

Primary enterolithiasis is a rare but important cause of small bowel obstruction and should be considered in patients presenting with recurrent or unexplained subacute intestinal obstructive symptoms. Localized intestinal stasis, even in the absence of classical risk factors, can generate a microenvironment conducive to stone formation. In our case, severe hypothyroidism with an associated stricture may have resulted in prolonged stasis and subsequent enterolith formation, causing obstruction. However, this association cannot be definitively established based on a single case and should be interpreted with caution. This case highlights the importance of maintaining a high level of suspicion, especially in at-risk patients such as those with severe hypothyroidism presenting with intermittent and partial obstructive symptoms. Early surgical intervention is the mainstay of treatment for larger stones, along with addressing the underlying pathology.
